# Impact of liquid antibacterial soap and hand sanitizer on DNA transfer in forensic investigations: an experimental study

**DOI:** 10.1093/fsr/owae068

**Published:** 2024-12-03

**Authors:** Francesco Sessa, Elisabetta Panepinto, Monica Salerno, Mario Chisari, Massimiliano Esposito, Giuseppe Cocimano, Cristoforo Pomara

**Affiliations:** Department of Medical, Surgical and Advanced Technologies “G.F. Ingrassia” University of Catania, Catania, Italy; Department of Medical, Surgical and Advanced Technologies “G.F. Ingrassia” University of Catania, Catania, Italy; Department of Medical, Surgical and Advanced Technologies “G.F. Ingrassia” University of Catania, Catania, Italy; “Rodolico-San Marco” Hospital, Catania, Italy; Faculty of Medicine and Surgery, “Kore” University of Enna, Cittadella Universitaria, Enna, Italy; Department of Mental and Physical Health and Preventive Medicine, University of Campania “Vanvitelli”, Napoli, Italy; Department of Medical, Surgical and Advanced Technologies “G.F. Ingrassia” University of Catania, Catania, Italy

**Keywords:** forensic sciences, touch DNA, hand sanitizer, forensic analysis, forensic implications, indirect DNA transfer

## Abstract

DNA transfer, whether intentional or not, is becoming an important part of forensic evidence gathering and analysis. This study seeks to determine the impact of liquid antibacterial soap and hand sanitizer use on the DNA present in the palms of hands that may potentially reduce direct transfer. Twelve volunteers were enrolled and typed. Afterwards, each palm was swabbed without considering previous activities to collect background DNA profiles. Subsequently, each subject washed his/her hands for 30 s with antibacterial soap first, and after with sanitizing gel and then air-dried them; after drying, each palm was immediately swabbed for DNA typing. The results of this study demonstrated that the possibility of recovering a complete profile from a hand swab is strictly related to previous activity: considering the results of the samples taken before handwashing, there is the possibility of having a median recovery of 80.01%, while it is very low (median recovery was 3.43%) after hand washing with antibacterial soap followed by alcohol-based hand sanitizer. Analyzing the results after handwashing, we were unable to detect any drop-in alleles. Moreover, we determined that in 11/12 samples, less than nine alleles were detected: considering that the kit used for the profiling could analyze 23 short tandem repeats (STRs), it is possible to conclude that we obtained inconclusive profiles. Based on the findings of the present study, it is more difficult to obtain a perpetrator’s profile if he/she used liquid antibacterial soap and hand sanitizer immediately before the criminal action.

## Introduction

In forensic investigations, the collection and examination of biological traces have provided a breakthrough in the identification process that makes it easy to identify guilty people and, at the same time, helps innocent people escape acquittal [1]. At the crime scene, it is possible to collect biological samples such as fresh blood or bloodstains, liquid saliva or saliva stains, liquid semen or dried semen stains, and skin cells [2]. Moreover, the “hand swab” can provide valuable genetic evidence in criminal investigations. This sampling could be performed both on the hands of the suspect and/or the victim, particularly in cases of physical assault or violence [3]. The genetic material recovered from hands is often composed of skin cells and sweat; however, it could include nonself DNA. The best way to collect this biological sample is using a moistened sterile swab, which is rubbed on the surface of the skin [4].

In the last few decades, the emergence of advanced profiling kits that are more sensitive and specific has led to resolving complicated or unsolved cases [5]. However, while the use of these technologies has expanded, in specific cases, there can be some challenges in interpreting genetic data: notably, the probability of finding a DNA profile from a minimal amount of DNA has significantly increased the possibility of finding nonrelated profiles at the crime scene [6]. Indeed, when a subject handles an object without gloves, DNA is released onto the surface of the object [7–10]. The so-called “touch DNA” is constituted of anucleate corneocytes, fragmentary cells/nuclei, nucleated epithelial cells from the hand, transferred nucleated cells, and cell-free DNA [11–13]. At a crime scene, it is possible to sample touched items and surfaces as sources of “touch DNA”, although it is challenging to understand the DNA transfer mechanisms by which individuals leave DNA behind. The capacity to release “touch DNA” may vary depending on the subject: after the first studies in this field, forensic experts concluded that a subject may be categorized as either a “good shedder” or a “poor/bad shedder”, based on the subject’s capacity to shed trace DNA [14, 15]. Subsequently, further studies clarified that there are three status categories to define the “shedder status”: high, middle, and low shedder [16, 17]. Furthermore, a recent research article indicates that further work may need to improve the current three-category classification in order to better define the shedder status in the forensic context [17]. The amount of DNA left behind is a vital parameter that impacts direct and indirect DNA transfer. In a recent research paper, Burrill et al*.* [18] demonstrated that a significant portion of DNA that could be collected on human hands comes from external sources rather than being produced by the hands themselves. This externally derived DNA is often from the same person, transferred from other body parts, and is acellular. Other authors confirmed that the amount of self-DNA deposited from hands is highly influenced by individual levels of accumulated facial DNA, and cells/DNA are often transferred to hands by touching or rubbing one’s face [14].

DNA transfer, whether intentional or not, is becoming an important part of forensic evidence gathering and analysis. It is well established that people can unconsciously transmit their DNA to several surfaces *via* direct and/or indirect contact [19–21]. Understanding factors affecting DNA transfer is very important since identification of a profile at a crime scene needs to be associated with the specific crime and additional evidence collected during the investigation. DNA transfer, persistence, prevalence, and recovery (DNA-TPPR) constitute the fundamental tenets of forensic DNA analysis. As previously described, transfer occurs when DNA is transferred directly through physical contact or indirectly *via* intermediaries, such as objects or surfaces, or by air [22, 23]. DNA persistence describes how long DNA remains detectable on a surface under various conditions, such as exposure to the environment, cleaning, or further contact. DNA prevalence represents the presence of cellular material, or DNA, of a person of interest on a surface or in the environment. Finally, DNA recovery is the process of collecting and analyzing DNA from various surfaces or samples [6, 24–26]. The successful recovery and analysis of DNA evidence are crucial for linking individuals with crime scenes, supporting or contradicting alibis as well as providing key evidence in criminal investigations and court proceedings.

Hygiene practices have recently increased by an exponential factor in the general population due to the COVID-19 pandemic that led to increased use of antibacterial liquid soaps and hand sanitizers [27]. These habits could greatly impact DNA transfer in real life and, as such, during crimes. Indeed, while their main purpose is to remove or minimize the number of microorganisms on the skin, forensic scientists are still interested in how hygiene products can affect DNA transfer and further analysis [28]. Because of the popularity of these products, it is crucial that their effect on DNA transfer in forensic investigations be completely understood.

This study seeks to determine the impact of liquid antibacterial soap and hand sanitizer use on the DNA present on the palm of hands that may potentially reduce direct transfer. Thus, this research aims to provide empirical evidence not only on the effectiveness of handwashing followed by sanitizer on the collection of DNA from hands but also on the impact on DNA transfer, filling a significant gap in our knowledge in this field of genetic investigations.

## Materials and methods

### Experimental model

The study was carried out following the Helsinki Declaration and was approved by the Ethics Committee of Catania (code 154/CEL). All participants signed informed consent and understood their right to withdraw at any time. Twelve volunteers (six males and six females), between the ages of 20 and 45, were enrolled. A buccal swab (Bioscientifica Srl, Rome, Italy) was performed for each participant to obtain their DNA reference profile.

Both hands were then swabbed, ignoring any previous activities in order to collect background DNA and/or other material. Using moderate pressure, a moistened swab was rolled over each hand covering the front and the back including all fingers. These samples were encoded using “HSWWXX” (Hand Swab Without Wash, Sample ID).

**Figure 1:**
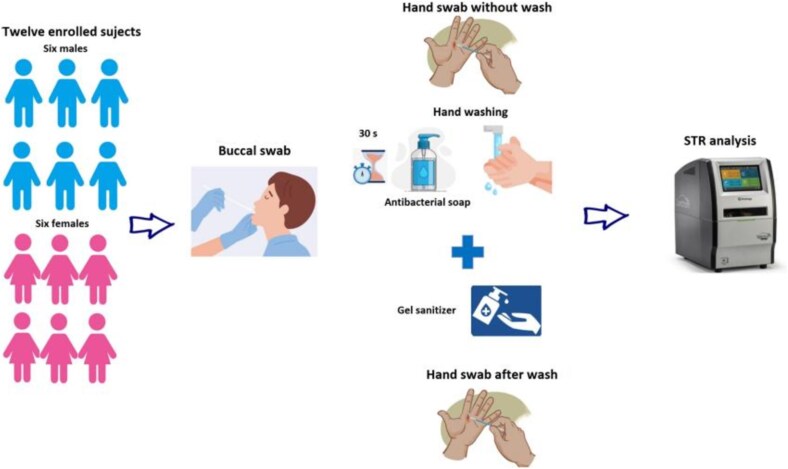
Twelve volunteers (six males and six females) were enrolled. One buccal swab for each volunteer was taken to obtain their reference profile. Both hands were then swabbed without washing first in order to collect a baseline sample. Then, each subject washed his/her hands for 30 s with antibacterial soap; moreover, each subject used a hand gel sanitizer and air-dried their hands. Finally, both hands were swabbed for each volunteer.

Subsequently, each subject washed his/her hands for 30 s with antibacterial soap first and after with sanitizing gel. After each step, the hands were allowed to be air-dried. At the end of this operation, both palms were immediately swabbed as previously described (“HSWAXX”, Hand Swab WAsh). All samples were collected using moistened sterile swabs for forensic use and certified DNA, DNase, RNase, and PCR inhibitor-free (Bioscientifica Srl). The experimental model is illustrated in Figure 1.

The ingredients of the antibacterial soap are water, cocamidopropyl betaine, sodium laureth sulfate, sodium chloride, cocamide diethanolamine, glyceryl oleate, coco-glucoside, perfume, benzalkonium chloride, styrene acrylates copolymer, benzyl alcohol, methylchloroisothiazolinone, methylisothiazolinone, and citric acid.

The hand sanitizer used in this study was composed of ethyl-denatured alcohol (572 g/L) and water.

For each sample, the operator wore double sterile gloves throughout the sampling process and when handling specimens (including exhibit bags) in order to minimize DNA transfer and contamination.

At the end of the sampling, 36 swabs were collected (12 buccal swabs, 12 HSWW, 12 HSWA). After DNA collection, the swabs were stored at −20°C until further processing.

### DNA extraction and quantification

Following the manufacturer’s instructions, DNA was extracted from the samples taken from the hands using a commercial silica-based, QIAmp DNA Investigator Kit (Qiagen, Hilden, Germany), with a final elution volume of 50 μL. Buccal swabs were analyzed with the KingFisher Duo Prime (Life Technologies), using the Magmax DNA Ultra 2.0 Kit (Life Technologies, Carlsbad, CA, USA), with a final elution volume of 60 μL. For each extraction session, a negative control was used.

Total DNA quantification was performed using the NanoDrop spectrophotometry (Life Technologies) and Qubit dsDNA Assay Kit along with the Qubit Fluorometer 3.0 (Life Technologies), according to the manufacturer’s protocol. The NanoDrop allowed to obtain a preliminary DNA quantification that was functional to the choice of Qubit Assay Kit (High Sensitivity kit for initial DNA sample concentrations of 5 pg/μL–120 ng/μL or Broad Range kit for initial DNA sample concentrations of 0.2–4 000 ng/μL).

### DNA profiling

Based on Qubit quantification, Promega PowerPlex® Fusion 6C System (Promega Corporation, Madison WI, USA) was used to obtain the DNA profiling: this kit amplifies and characterizes 23 autosomal STRs, three Y-STRs, and the amelogenin gene in 1 ng of DNA, according to the manufacturer’s protocol. In particular, each profile is composed of six panels: panel A (peaks of the FL-6C-labeled loci: amelogenin, D3S1358, D1S1656, D2S441, D10S1248, D13S317, and Penta E), panel B (peaks of the JOE-6C-labeled loci: D16S539, D18S51, D2S1338, CSF1PO, and Penta D), panel C (peaks of the TMR-6C-labeled loci: TH01, vWA, D21S11, D7S820, D5S818, and TPOX), panel D (peaks of the CXR-6C-labeled loci: D8S1179, D12S391, D19S433, SE33, and D22S1045), panel E (peaks of the TOM-6C-labeled loci: DYS391, FGA, DYS576, and DYS570), and panel F that shows the electropherogram of internal lane standard (WEN 500).

Amplification was performed on the Veriti™ 96-well Thermal Cycler (Applied Biosystems, Foster City, CA, USA) instrument. Fragment analysis was carried out on the Spectrum Compact CE System (Promega Corporation), and each run was analyzed with GeneMarker HID V3.2.0 for Spectrum CE Systems (Promega Corporation). The allelic ladder, positive control, and negative controls (both for DNA extraction and for DNA amplification) were run at the same time.

### Data interpretation

During the analysis, we obtained inconclusive profiles, which had fewer than 10 typed loci, single source profiles, and mixed profiles, which displayed more than two allelic signals at two or more loci. When one or two alleles at each locus had a peak height ratio of at least 3:1 in relation to the other alleles of the same locus, the mixed profiles were categorized as having a strong contributor to the overall profile results [29, 30]. The generated DNA profiles from hands (both “HSWW” and “HSWA”) were compared with the reference samples of the donors from the buccal swab, counting the number of alleles, drop-in alleles (presence of alleles in the obtained profile that are not present in the reference DNA profile), and drop-out alleles (absence of alleles in the obtained profile that are present in the reference DNA profile). The amelogenin gene alleles were excluded from the counting, evaluating only the 23 autosomal STRs. The number of alleles counted in the reference profile obtained from the buccal swab was considered the number of expected alleles. The number of detected alleles in samples obtained from hands before washing (HSWW) and after washing (HSWA) was compared with the relevant reference samples, and the results were expressed as a percentage.

### Statistical analysis

Descriptive statistical analyses were performed using Microsoft Office Excel 2007 (IBM Corp., Armonk, NY, USA), Stata/IC (Stata Corp LLC, College Station, TX, USA), and R (https://cran.r-project.org). The Kolmogorov–Smirnov test was used to determine the normality of data distribution. All variables were normally distributed. Student’s *t*-test was used for comparisons between two groups. *P* < 0.05 was considered to represent a statistically significant difference.

## Results

### DNA profile analysis

The number of predicted alleles was determined by counting the number of alleles (excluding sex alleles) in the reference profile that was obtained from the buccal swab. Samples taken from the hands before and after washing (HSWW and HSWA) were compared with relevant reference samples, counting the number of detected alleles with the exclusion of amelogenin gene STRs. Moreover, the number of drop-in and drop-out alleles was counted. All data are summarized in Table 1.

**Table 1:** Summary of the main data obtained after DNA profile analysis.

Sample ID	Number of alleles		
	Reference profiles	HSWW/drop-in	HSWA/drop-in
M1	42	34/6	15/0
M2	44	43/1	2/0
M3	41	41/36	6/0
M4	43	13/2	0/0
M5	45	45/0	9/0
M6	43	34/6	1/0
F1	38	35/4	2/0
F2	36	13/1	0/0
F3	45	40/0	1/0
F4	40	17/0	0/0
F5	45	11/0	4/0
F6	42	18/2	0/0

M: male; F: female; HSWW: hand swab without wash; HSWA: hand swab wash.

**Table 2:** Summary of the detected alleles after handwashing.

LOCUS	M1	M2	M3	M4	M5	M6	F1	F2	F3	F4	F5	F6
D3S1358	0	0	0	0	0	0	0	0	0	0	0	0
D1S1656	0	1	0	0	0	0	0	0	0	0	0	0
D2S441	0.5	0	0	0	0.5	0	0	0	0	0	0.5	0
D10S1248	0	0	0	0	0.5	0	0.5	0	0	0	0	0
D13S317	0	0.5	0	0	0	0	0	0	0.5	0	0	0
Penta E	1	0	0	0	0	0	0	0	0	0	0	0
D16S539	1	0	0	0	0	0	0	0	0	0	0	0
D18S51	0	0	0.5	0	1	0	0.5	0	0	0	0	0
D2S1338	0	0	0	0	0.5	0	0	0	0	0	0	0
CSF1PO	0	0	1	0	0	0	0	0	0	0	0	0
Penta D	0	0	0	0	0	0	0	0	0	0	0	0
TH01	0.5	0	0	0	0	0	0	0	0	0	0	0
vWA	1	0	0	0	1	0	0	0	0	0	0	0
D21S11	0	0	1	0	0.5	0	0	0	0	0	0	0
D7S820	0	0	0.5	0	1	0	0	0	0	0	0	0
D5S818	1	0	0	0	0	0	0	0	0	0	0	0
TPOX	0.5	0	0	0	0.5	0	0	0	0	0	0	0
D8S1179	1	0	0	0	0	0	0	0	0	0	0	0
D12S391	0.5	0	0.5	0	0	0.5	0	0	0	0	1	0
D19S433	0	0	0	0	0	0	0	0	0	0	0	0
SE33	0	0	0	0	0	0	0	0	0	0	0	0
D22S1045	1	0	0	0	0	0	0	0	0	0	0	0
DYS391	0	0	0	0	0	0	–	–	–	–	–	–
FGA	0.5	0	0	0	0	0	0	0	0	0	0.5	0
DYS576	0	0	0	0	0	0	–	–	–	–	–	–
DYS570	1	0	0	0	0	0	–	–	–	–	–	–

From the analysis of the obtained profiles, we observed a high recovery of alleles from the hand swab before handwashing: as summarized in Table 1, we obtained a complete profile in 2/12 samples. It is important to consider that the swabs taken before handwashing were done without knowing the activity before sampling: this choice for our experimental model was taken to reproduce the variability of real cases, where we do not know the activities performed by the victim, or the perpetrator, before the crime. Indeed, the status of the DNA presence on the hands for each subject during daily activities is usually unknown. The main goal of this experimental study was to test the effects of the combined use of soap and sanitizer on the possibility of recovering DNA from hands. Considering the detection of the nonself-alleles (drop-in), we found a very limited number of external alleles in 11/12 samples, ranging from zero (4 samples) to six alleles. Only for 1/12 sample (M3) did we detect a mixture of two subjects, determining that the major contributor was the subject involved in the experiment. The external profile belonged to an unknown person who was more likely in social contact with M3 before the sampling.

Analyzing data obtained from the swabs taken after handwashing and sanitizer use, we were unable to find any alleles in 4/12 samples, while in 5/12, we found <5 alleles, in 1/12, we detected 6 alleles, in another, 1/12, 9 alleles, and finally, only in 1/12 did we detect 15 alleles. No drop-in alleles were found, despite the large number of profiles that could be considered inconclusive. We analyzed each locus of the detected alleles after handwashing, giving a value of 1 when we were able to detect both alleles or one allele in the case of homozygous, 0.5 when we detected only one allele in heterozygous, and a value of 0 if we are unable to detect any alleles. The data are summarized in Table 2.

Analyzing the data reported in Table 2, it is possible to conclude that we found only one allele in the Y-STRs. Considering the autosomal loci, it is possible to conclude that there is no correlation between the dimension of the amplification and the possibility of finding alleles: after handwashing, allele detection was a rare event.

**Figure 2:**
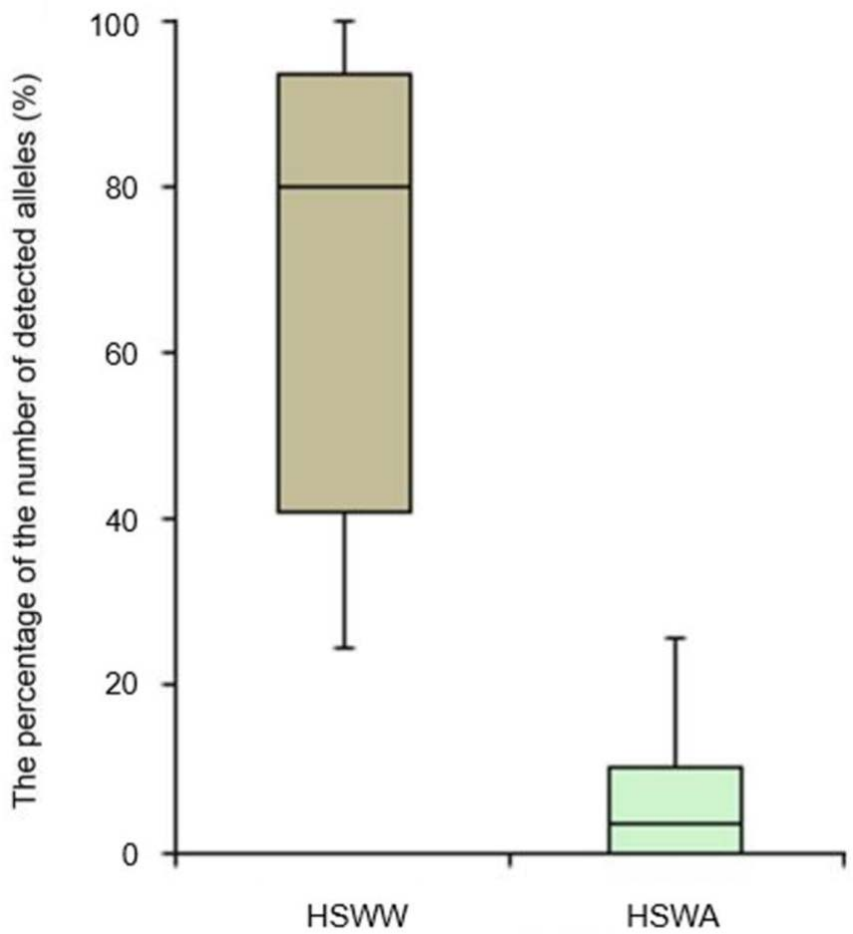
The box plot analysis compares the percentage of the number of detected alleles in the samples taken from the hands before washing (HSWW) group *vs.* the samples taken from the hands after washing (HSWA) group.

Moreover, we further analyzed the data, summarizing them as percentages by box plot analysis (Figure 2).

As highlighted in the graphic representation, this study demonstrated that the percentage of detected alleles is higher in the HSWW group (median recovery was 80.01%) compared with the HSWA group (median recovery was 3.43%). There were statistically significant differences between the two groups as determined by Student’s *t*-test [*P* <0.05(1.31834 × 10^−5^)].

**Figure 3:**
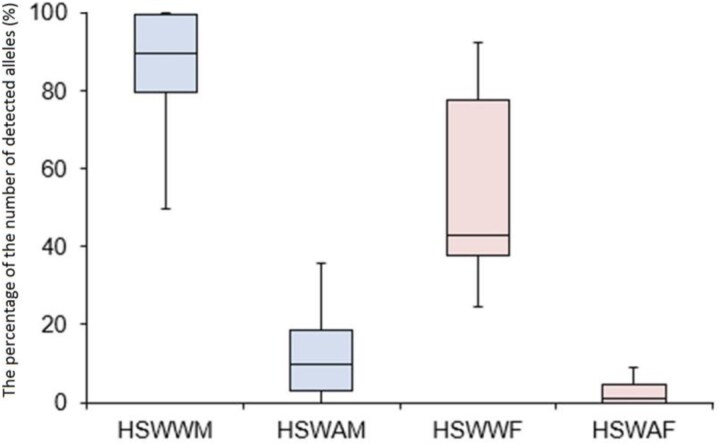
The box plot analysis compares the percentage of the number of detected alleles in samples subdivided under sex criterion: male (HSWWM group vs. HSWAM group) and female (HSWWF group *vs.* HSWAF group). Moreover, we compared data of the allele recovery before handwashing (HSWWM group *vs.* HSWWF group) and after handwashing (HSWAM group *vs.* HSWAF group). HSWW: hand swab without wash; HSWA: hand swab wash.

Subsequently, we analyzed the data under the sex criterion, summarizing the percentage data by box plot analysis (Figure 3).

Based on the sex criterion, the results remained the same: the percentage of detected alleles from the hand swab was higher in the groups before handwashing compared with the groups after the use of liquid antibacterial soap followed by hand sanitizer use, both for males (HSWWM group *vs.* HSWAM group, *P* <0.05, 0.000826961) and for females (HSWWF group *vs.* HASWAF group, *P* <0.05, 0.007140599). Comparing the allele recovery before and after handwashing, we compared male and female groups: we did not find statistically significant differences before handwashing (HSWWM *vs.* HSWWF, *P* >0.05), although the median value was higher in the male group (median = 84.34%) compared with the female group (median = 42.67%). No statistical differences were found comparing the alleles recovered after handwashing under the sex criterion (HSWAM *vs.* HSWAF, *P* >0.05). However, the median recovery was higher in the HSWAM group (median = 9.58%) compared with the HASWAF group (median = 1.11%).

## Discussion

When investigating a crime scene, the genetic material collected from the hands of individuals present at the scene can provide crucial evidence [31]. Indeed, in cases of physical assault or violence, genetic material obtained from the hands of the victim, or the perpetrator, can aid in identifying the parties involved, helping to determine the course of legal action and establishing the sequence of events during the assault [32]. The results of this research provide important information about the possibility of recovering DNA from hands using a swab before and after handwashing: in this way, this article clarifies several important aspects concerning the possibility of collecting hand swabs for forensic purposes [4, 12, 33, 34]. In our experimental model, we combined the use of soap and sanitizer because their combined use has become common practice in the postpandemic period. Moreover, Vindenes et al*.* [35] investigated the effects of alcohol-based hand sanitizer and hand washing with soap and water on the composition of bacterial skin microbiota. In the forensic field, various studies have explored the effects of handwashing or sanitizer individually [18, 28]. To the best of our knowledge, this is the first study to test the direct impact of their combined use on DNA recovery from hands. As previously described, the possibility of recovering a complete profile from a hand swab is strictly related to previous activity: considering the results of the samples taken before handwashing, there is the possibility of having a median recovery of 80.01%. These data confirmed the possibility of obtaining an interpretable or complete profile from this kind of sampling. Despite the fact that the possibility of recovering a complete profile is higher in male samples compared with female samples, there were no statistically significant differences. As previously described, these results could be related to shedder status [4]: several authors concluded that it could also be related to gender and previous activities [14]. Individual differences in shedder status are commonly acknowledged; however, it is unclear if these differences result from physiological processes, individual behaviors, or other external circumstances [14]. Today, it is possible to classify a subject in one of three statuses: high, intermediate, and low shedder [17, 20, 36]. The results of the present study are in agreement with these studies, highlighting the inter- and intra-individual variability and confirming the importance of previous activities in order to recover a DNA profile from hand swabs.

Another important consideration is the detection of the nonself-alleles (drop-in), only in one case did we obtain a complete mixture, even if the major contributor was the subject involved in the experiment. Obviously, the other profile belonged to an unknown person who probably had social contact with the subject before sampling. The low number of drop-in alleles in other samples could be related to the activities performed before swabbing: for example, the use of nonpersonal items such as a keyboard, cellphone, mouse, and others [33, 37]. These data are in agreement with a previous study: Jansson et al*.* [14] reported that in their experimental model, only a few samples contained foreign DNA.

Analyzing the results after handwashing it is possible to conclude that when this operation is performed before sampling, it reduces the possibility of collecting the nonself-DNA: in all samples, we were unable to detect any drop-in alleles. Moreover, we determined that in 11/12 samples, less than nine alleles were detected: considering that the kit used for the profiling could analyze 23 STRs, it is possible to conclude that we obtained inconclusive profiles. Only in 1/12 sample did we detect 15/42 alleles: considering the high number of nondetected alleles (27) also in this case, we obtained an incomplete profile, which could be linked with difficulty if found in a real case. In this regard, despite the kit that was used could amplify three Y-STRs, we were unable to detect all Y alleles (we detected only one out of three Y-STRs). Moreover, in all other male samples, we were unable to detect the three Y-STRs: these results highlighted the difficulty of relating the obtained partial profile to a reference profile if we had obtained the same results in a real crime scene investigation. Another important consideration is that the obtained results are not related to sex: we did not find statistically significant differences comparing male *vs.* female results, although males transferred more DNA compared with females. These results are in agreement with previous studies: it has been demonstrated that males secrete much more face sebum than women do, which might provide a scientific explanation for why it is possible to collect more DNA from male hands [38]; in the same way, a male could more likely transfer his DNA to an object, increasing the possibility of generating a transfer.

The findings of the present study could provide important insights into the evaluation of DNA transfer. Indeed, the analysis of genetic material from the hands of individuals can provide information concerning any contact and interactions between individuals or in the management of objects. Based on the data of the present experimental paper, considering that we obtained an inconclusive profile from all analyzed samples recovered after handwashing, it is possible to hypothesize that the use of antibacterial soap, followed by sanitizer liquid, can notably reduce the possibility of a direct transfer of touch DNA. Since liquid antibacterial soaps and hand sanitizers form an important part of common hygiene practices, particularly after the pandemic period, the potential impact that they may have on DNA transfer significantly contributes to assessing the weight associated with DNA evidence. As previously described, the median recovery was 3.43% in the HSWA group. Comparing the data after handwashing under the sex criterion (HSWAM *vs.* HSWAF, *P*-value >0.05), no statistical differences were found. These results are in part in contrast with the increased DNA shedding propensity of male donors reported by other authors [39].

The results of the present study could be considered in the formulation of the Bayesian model in the interpretation of touch DNA transfer because it offers a statistical framework for determining the likelihood of such transfers in the forensic evaluation of the weight of evidence. The use of prior probabilities, which reflect the starting assumptions about the likelihood of various events, is a crucial component of the Bayesian model. The assumptions and input data of the Bayesian model are critical: erroneous assumptions, biases, or inaccuracies in the data may affect how reliable the evidence evaluation is; in this way, the use of artificial intelligence technologies could be helpful in the future [40]. The importance of DNA transfer and the influence of the combined use of liquid antibacterial soaps and hand sanitizers is crucial for the accurate evaluation of evidence recovered at crime scenes, improving the interpretation of forensic investigations. A thorough knowledge of DNA-TPPR could be very helpful for a proper interpretation of DNA evidence, in establishing associations between individuals and crime scenes as well as ensuring that forensic analyses are carried out with integrity.

This study has several strengths. Firstly, the sample size includes an equal number of males and females, allowing for an evaluation of differences based on sex. Additionally, each DNA profile was obtained using a kit that amplifies 23 autosomal STRs, three Y-STRs, and amelogenin.

However, there are several limitations to consider. The number of enrolled subjects is relatively small (12 in total, with six males and six females). The study evaluates the combined use of soap and sanitizer but does not assess the individual effects of water, soap, or sanitizer alone.

Lastly, regarding the baseline swab, our experimental model chose to perform the baseline swab (hand swab before handwashing) without knowing the previous activities of each subject. This choice could be seen as both a strength—since it mirrors real-life situations in forensic investigations where the previous activities of victims or suspects are unknown—and a limitation, as it prevents a complete evaluation of the results.

## Conclusions

Based on the findings of the present study, it is more difficult to obtain a perpetrator’s profile if he/she used liquid antibacterial soap and hand sanitizer immediately before the criminal action. As previously described, less DNA will be recovered from touched surfaces if they wash their hands before committing a crime. Nevertheless, an STR typing study after handwashing and sanitizer use would still be feasible and might help identify the offender if this does not fall below a specific threshold. Moreover, future studies could be important in order to clarify if after a period of time (i.e. 1 h from handwashing) and after various activities, both self- and nonself-DNA might be present on the handled object.
